# Walking With Meaning: Subjective Experiences of Physical Activity in Dementia

**DOI:** 10.1177/2333393615605116

**Published:** 2015-09-30

**Authors:** Jennifer McDuff, Alison Phinney

**Affiliations:** 1 Fraser Health Authority, Surrey, British Columbia, Canada; 2 University of British Columbia, Vancouver, British Columbia, Canada

**Keywords:** dementia, exercise/physical activity, older people, interpretive description

## Abstract

Physical activity is beneficial for people with dementia, but little research explores subjective experiences of physical activity in this population. Interpretive description guided the analysis of 26 interviews conducted with 12 people with dementia. Three themes described the subjective meaning of everyday physical activity: Participants were *attracted to activity* because it improved physical well-being, provided social connections, gave opportunity to be in nature, and provided structure and focus; participants experienced *impediments to activity* because of physical discomfort, environmental factors, lack of enthusiasm, and memory loss; and participants *made adjustments* by choosing walking over other activities and by being active with others. Results show that physical activity remains important for people with dementia, although they encounter barriers. They may prefer walking with others as a form of activity. Findings could influence how nurses conceptualize wandering and suggest that walking programs could be well received by people with dementia.

## Introduction

Increasing physical activity is recommended as an effective intervention for enhancing health and quality of life for older people. Research shows that exercise reduces risk for disease, enhances function, and prevents cognitive decline as people age. These benefits are known to extend to people with dementia, although there is limited research on how to promote physical activity in this group. In particular, we know little about how people with dementia perceive the significance of physical activity in the context of everyday life. The purpose of this study was to contribute new knowledge in this area, with an ultimate aim of better understanding how to support people with dementia to be more active.

### Benefits of Physical Activity for Older People

In this study, the term *physical activity* refers not only to exercise and sporting activities but also to everyday pastimes that have a significant physical component (e.g., gardening, walking, or household chores). There is a growing body of research on physical activity for older people, with a number of systematic reviews and meta-analyses showing numerous benefits (Taylor et al., 2004). In particular, older people who are active have lower rates of cardiovascular disease, diabetes, and cancer (Warburton, Nicol, & Bredin, 2006). They may also experience a range of improved health outcomes including reduced falls (Sherrington et al., 2008) and improved mental health and well-being (Lindwall, Rennemark, Halling, Berglund, & Hassmén, 2007; Netz, Wu, Becker, & Tenenbaum, 2005). These and other reviews have supported the development of evidence-based guidelines for physical activity of older people (e.g., World Health Organization, 2010).

There is growing interest in the benefits of physical activity for cognition in older people. Numerous studies have shown that people who are more physically active are less likely to develop cognitive impairment than those who are less active (Etgen et al., 2010; Hamer & Chida, 2009), and in a classic study conducted by Colcombe and Kramer (2003), a meta-analysis showed that aerobic training can even improve cognition in healthy older adults. More recent research suggests that this may also hold true for those with neurocognitive deficits who are at increased risk for developing dementia (Baker et al., 2010; Lautenschlager et al., 2008; Nagamatsu, Handy, Hsu, Voss, & Liu-Ambrose, 2012).

### Factors Influencing Physical Activity of Older People

Despite the known benefits of physical activity for older people, this age group remains one of the most sedentary (Sun, Norman, & While, 2013). Barriers to physical activity among older people include perceived lack of time or interest, having no one to exercise with, and the belief that exercise is unsafe or uncomfortable (Brawley, Rejeski, & King, 2003; Mathews et al., 2010; Moschny, Platen, Klaaßen-Mielke, Trampisch, & Hinrichs, 2011). People with dementia may face additional barriers, including mobility impairment or cognitive decline. However, there has been little research in this area, and it is unclear how these changes influence their experience of physical activity.

Several studies have identified the importance of social support in promoting physical activity. Older people are more likely to be active if they have social networks that encourage activity and provide instrumental support such as transportation and other forms of access (Mathews et al., 2010; Schutzer & Graves, 2004).

### Physical Activity for People With Dementia

Although the issue of physical activity for older people has received considerable attention in the research literature, it is only recently that people with dementia have been included. The number of studies remains small, but recent reviews have provided evidence that physical activity can improve well-being and mobility, and slow the rate of functional decline of people with dementia (Littbrand, Stenvall, & Rosendahl, 2011; Pitkälä, Savikko, Poysti, Strandberg, & Laakkonen, 2013; Potter, Ellard, Rees, & Thorogood, 2011). This suggests that physical activity could be a useful intervention, and indeed, recent randomized controlled trials have shown that exercise programs for people with dementia can have beneficial effects for their ability to complete activities of daily living (Forbes, Thiessen, Blake, Forbes, & Forbes, 2015). These groups may also be enjoyable. Stathi and Simey (2007) found that nursing home residents who took part in a 6-month exercise intervention appreciated the social connections within the group and felt physically stronger and more confident by the end of the program.

Apart from this one study, the subjective significance of physical activity for people with dementia has not received much attention in the research literature. This is an important gap. The design of programs and services for people with dementia should be informed by evidence about the views and perceptions of people with dementia themselves (Goldsmith, 1996; van Baalen, Vingerhoets, Sixma, & de Lange, 2010). In recognition of this, there is a growing body of research with the aim to examine and better understand the subjective experiences of people with dementia (van der Roest et al., 2007; von Kutzleben, Schmid, Halek, Holle, & Bartholomeyczik, 2012). A few studies focusing on activity in general have found that people with dementia may still enjoy exercise and recreation, and in fact, activities they previously took for granted may become more important in their daily life (Menne, Johnson, Whitlatch, & Schwartz, 2012; Phinney, Chaudhury, & O’Connor, 2007). While some report doing less than before, others find ways to modify their involvement so they can remain active and maintain a sense of continuity (Menne, Kinney, & Morhardt, 2002; Phinney et al., 2007; Phinney, Dahlke, & Purves, 2013). These studies have provided hints as to the importance of physical activity from the perspective of people with dementia, but further research is necessary to address this question directly. Therefore, the purpose of this study was to seek these perspectives that could ultimately be used as evidence to inform practice in this area.

## Method

This article reports findings from a qualitative study that sought to explore the meaning of activity in the everyday lives of people living with dementia. Early analysis showed that exercise and other forms of physical activity were important to many of the participants with the topic coming up spontaneously in many of the interviews. Therefore, an in-depth analysis was done using a subset of interview data to answer the following research question:

**Research Question:** What does involvement in physical activity mean for people with dementia?

### Research Design

The study was conducted in two phases: Phase I included 10 participants recently diagnosed (<4 months) at a specialty memory clinic, while Phase II included 20 participants living in the community who had been diagnosed with dementia for at least 1 year. Beyond their diagnosis, inclusion criteria were broad to ensure as diverse a sample as possible; participants had to be able to communicate in English and able to provide informed consent.

Data were gathered on multiple occasions with each person being interviewed an average of 4 times over a period of several months (including individual and family interviews). These conversational style interviews lasted an average of 45 to 60 minutes and were conducted in the person’s home to obtain an in-depth understanding of their activity in its everyday context. Interviews began with an invitation to the person to talk about the activities that were important in their daily lives. The interviewer would follow up with gentle probes to encourage the person to say more, being alert to how the person described what supported their involvement in the activity and what made it difficult. When the person had trouble remembering or describing details, the interviewer would invite them to describe their feelings about the activity. When family members were present, they would sometimes provide additional details, in which case, the interviewer would follow up to confirm with the person with dementia. This was in an effort to ensure that the data would be as close as possible to the person’s experience rather than becoming a “proxy report.”

The research received approval from the university’s ethics review board, and all participants provided informed consent. Because these participants had varying degrees of cognitive impairment, particular care was taken with the consent process. The interviewer took time to discuss the study at the outset, ensuring that the participant could describe the study procedures in his or her own words and express understanding that participation was voluntary and it was possible to withdraw at any time. Following the “process consent method” as described by Dewing (2007), verbal consent was renewed on each occasion and as needed throughout the interviews. As a further step to protect participants, identifying details in the data were changed to help ensure anonymity.

### Sample

For this analysis, a sub-sample of 12 participants with dementia was identified as those who talked about their involvement in physical activity. They were between the ages of 65 and 86 years. There were equal numbers of men and women, and equal numbers from Phase I (diagnosed less than 4 months) and Phase II (diagnosed between 1 and 3 years), thus reflecting a range of impairment from mild to moderate. All the participants came from a middle class background and identified themselves as being in good overall physical health. Eight were living with a spouse and 4 were living alone, either in their own home (*n* = 10) or in an assisted living residence (*n* = 2). Thus, while the sample for this analysis was homogeneous in terms of social background and physical health, it was diverse in terms of age, gender, degree of cognitive impairment, and living situation.

### Data Analysis

A total of 26 interviews that included discussion of physical activity by the person with dementia were identified for analysis. Transcripts were analyzed for themes to identify what daily physical activities the participants were involved in and how they described the significance of this in their lives.

As an applied qualitative methodology, interpretive description was a suitable choice to guide this analysis. The purpose here was ultimately a practical one: to explore subjective experiences of physical activity to better understand how to support people with dementia to be more active. This aligns closely with the goals of interpretive description, where analysis is to “capture themes and patterns within subjective perceptions” to develop findings that are “capable of informing clinical understanding” (Thorne, Reimer-Kirkham, & O’Flynn-Magee, 2004, p. 3).

As first author, McDuff took the lead on the data analysis and followed an interpretive description approach as described by Thorne (2008). This began with a careful in-depth read of all the transcripts. After the initial read through, the transcripts were read again and preliminary coding, staying close to the data, was started. As the coding scheme developed, patterns of relationship between and across the codes were identified, and these relationships were developed into larger categories. From these categories, analysis moved to the development of themes and sub-themes. This was not a simple linear process of increasing abstraction but rather involved multiple iterations back and forth between the emerging interpretations and the coded data as a way of exploring and testing out new ideas. Credibility was further enhanced through meeting at regular intervals as a research team to discuss, challenge, and further develop the emerging interpretations. These analytic processes, along with the incorporation of verbatim quotes as support for the themes and sub-themes, helped create a coherent and resonant account of the experiences and meaning of physical activity for people with dementia.

## Findings

The findings of this study are organized thematically in three categories. All the participants spoke of activity as something they desired and enjoyed (*Attractions of Activity*), but at the same time, it was more difficult and less frequent in their lives (*Impediments to Activity*). To remain active, they found ways to do things differently *(Making Adjustments).*

### Attractions of Activity

Participants were involved in different types of physical activity, including walking, exercise classes, housework, gardening, and swimming. Throughout the interviews, they spoke often about why these activities were important to them.

#### Physical well-being

Everyone described that being physically active made them feel better. Several spoke about how exercising was important because it kept them “healthy” and “fit.” One participant described her water aerobic class, “It’s in the water and you’re into chest level and you’re running back and forth and doing all this exercise and all this kind of, it’s really good for you.” Another talked of daily walks, “I think that’s what keeps me fit. And really really healthy.”

Some wanted to exercise so they could still feel and look young, while others found that activity allowed them to manage physical problems such as constipation, weight gain, and joint pain. One woman talked about the range of benefits she received from her daily 2- to 3-hour walk at a nearby park: “I have rheumatoid arthritis and the walking helps keep my joints in better shape and I’m hoping it’s keeping my weight down. And it keeps me, I mean, I’m 77 but most people think I am in my 60’s.”

#### Social connections

Beyond the physical benefits, the most common attraction to being active was the pleasure people experienced from the social connections it provided. When participants had someone join them, they seemed to walk further or exercise more because they enjoyed the social side of the activity. When one woman was asked about her very active routine, she explained, “Well I enjoy it too, here out with other people, you know, doing stuff.” Those who took an exercise class drew pleasure from the people in the class, their relationship with the instructor, or the social activity they took part in after the class. “It’s very nice to have a companion” one of the participants said of taking an exercise class. Another described walking back and forth to a coffee shop with her daughter, saying “It’s nicer if there’s a couple.”

#### Being out in nature

Being physically active was important for several participants because it provided opportunity to be outside in the natural world. One participant talked about enjoying being out and seeing the changing of seasons, while others spoke of how they appreciated being out in the weather, whether it was raining or the sun was shining. Several talked of walking in parks, along trails where they could enjoy nature, hearing the birds, and seeing trees and flowers. Some found particular pleasure in the solitude they experienced being outside on their own, walking on their own through neighborhoods, and enjoying the local scenery. In describing her frequent walks, one woman said, “It’s a beautiful area and I do enjoy it.”

#### Structure and focus

Part of the attraction of physical activity was that it helped people occupy their time when there was little else they could do. As one participant stated, “I do a lot of exercise and enjoy it and I’ve got nothing else to do with my day.” Another participant resorted to walking because he could not drive and could not identify any alternatives, saying “well, what else you doing ya know?” A husband in describing his wife’s daily activities said, “well there is nothing really” except when they would sometimes “go for a little walk around the neighborhood.”

It was common for participants to not know what to do with their time, but a dog to be walked or a spouse saying “let’s go for a walk” provided a desirable focus to their day. People described how much they enjoyed these outings; it made them feel like they were doing something and gave them a purpose to their day. One man looked forward to his weekly exercise class, describing what the class entailed:

We have two things in the morning. But first we have some kind of thing they teach you because they have things they teach you about and that, and then they have an exercise. And then you have lunch and then you go . . . do some other things out there.

Although it was difficult for him to describe the details of his activity, it was evident from his words that he valued the structure it brought to his day.

### Impediments to Activity

Participants in this study described a change in the frequency and level of activity from their younger years, and they had various understandings as to why they were not as active as they had been in their youth.

#### Physical discomfort

Participants talked about physical complaints as reasons for being less active; this included having sore legs, fatigue, and incontinence. For some, this meant that they were less able to sustain activity.

I don’t go for a walk as long or as much as I have been doing because it bothers me. And then I walk for a while and my legs get tired, then I come back in and sit down or lay down.

For others, physical discomfort prevented them from initiating activity in the first place. “With my arms and legs bothering me, I just don’t push it too far. I figure I might as well go to sleep and forget it.”

#### Environmental factors

Characteristics of communities, such as accessibility of parks and trails, poor sidewalk design, concrete roads that were difficult to walk on, and the quality of the environment such as poor weather conditions (rain, snow, and cold), played a role in influencing participants’ level of physical activity. One participant used to trail walk but because she no longer drives and walking on concrete physically bothers her, she does not walk as often anymore.

I go out for walks and I can walk quite a distance but, I notice that when you’re walking around concrete it’s a different story . . . it is hard on your feet . . . that’s why I used to like go out rock hunting. Because you’re, we were walking on rocks but that’s different! [Laughs]. . . . But ah, I don’t get out there anymore because I don’t have the car.

A participant stated that weather conditions determined whether he would go walking, “well depending on the weather, the weather’s been great really for walking. There’s been very little to stop us.”

#### Lack of enthusiasm

Several participants felt they were less physically active than before because they were feeling less ambitious and were less interested. One participant described it as being “not motivated,” while another explained how she was “finding it hard to set aside certain periods” for activity. One man talked of how he felt that with aging he had less energy than he had in his youth. He stated, “I feel like a clock. I feel like I gotta be wound up again. I need a shot of adrenaline or something.” As much as people spoke of activity as something they enjoyed, many found it hard to get started and did not have the mental or physical energy they had once had.

#### Memory loss

Many of the participants felt that their memory problems interfered with activities they used to enjoy, such as sports, swimming, or cycling. As one man explained, “I got these memory problems, it’s been harder for me to try and remember these things so I’m not as active as I was.” He had been an active gardener in the past, but was no longer active in these areas, wondering whether it was because “maybe I’m losing some of my smarts.” Other activities were no longer available because the participants (or their family) felt the activity was unsafe. Another man used to enjoy daily bike rides, but he is not doing that anymore, in part because he and his wife were both concerned that he would lose his way. Others were discouraged from engaging in household chores because of concerns they would do something dangerous.

### Making Adjustments

To remain active, people had to overcome these impediments, which was often difficult to achieve. Some discussed feeling frustration or regret with their decreased activity, but they said that they were “coping with it” and were “focused in other ways now.” They made adjustments to activity routines when these previous activities were too challenging to continue. “Sometimes you know your mind forces you in different directions so I cope with it as best I can.” Many of the participants simplified their activity routines to include walking, which was something they could easily do on their own. They also took advantage of social supports, finding ways to engage in activities with a companion.

#### Walking instead

Many of the participants had eliminated certain activities and turned to walking as an alternative. For example, one participant was a member of a community center where he sometimes attended exercise classes. He found the classes regimented, however, and talked about “missing out on things.” He did not go as often anymore but still walked back and forth to the community center for social events.

Walking was something that everyone felt confident to do because it was simple and they had been doing it all their lives. “Well, I don’t know. Just, I just seem to, I like walkin’. Why? Just it’s something you more or less do. It’s just, you know, you walk all your life.” They identified it as an ordinary activity that everyone does and appreciated that it could still be a part of their daily routine. “I walk everyday, rain or shine, the weather never deters me from walking.” But for many, it was also necessary; when they could not drive anymore, walking was sometimes the most ready alternative. For example, after losing his license, one man began walking to nearby shops rather than driving to the large malls on the edge of town. “Most of what I need to do I can do by walking downtown and the walking is good for me anyway.” When asked if not driving has affected his life, another man felt that being unable to drive had not really affected his life because now he walks daily to the places he needs to go. “Quite often I go for a long walk, you know where central valley is down here and the clubhouse and all that, well I go down there and around that and back, so that takes an hour or two.”

#### Having companionship

Whereas in the past, people had been able to be involved in physical activity on their own, increasingly they had to rely on others for support. One woman had been very involved in aerobics in her earlier adult years. She spoke of how she “used to be able to go” to a class, but now she had to rely on her husband to help her remain active; when he was able, he would come home from work mid-day and they would walk around the neighborhood together. Another participant and his wife had a paper route as a way to stay physically active. Delivering the newspapers in their neighborhood twice a week was a large focus in this man’s week, but he would not have been able to do it without the companionship of his wife.

Although doing activity with others brought a certain pleasure as described earlier, it could also be a source of some concern. One participant talked about the discomfort of having to depend on his friend to go with him to town: “Well my biggest concern is that I would rely too much on my friend and her automobile. And so I have to be very careful there. I don’t want to have to call on her too often.”

## Discussion

This research has shown that the meaning of physical activity for people with dementia is in some ways similar to its meaning for older people in general. It has also shown ways in which it is different, as people with dementia face unique barriers to which they adapt in particular ways. The parallels with existing research about physical activity of older people, and the contribution of these new findings about the meaning of physical activity for people with dementia, are further discussed below.

First and foremost, this research has shown that people with dementia continue to be attracted to physical activity; the notion of physically active citizenship was evident throughout the interviews. In this respect, the findings were similar to research conducted with older people without dementia. Like many older people, participants in this study were well aware of the societal message that “being fit” is important and believed that physical activity was good for their health (Crombie et al., 2004). They also appreciated the opportunities it afforded for social involvement. Older people are more likely to be active if they have someone to accompany them (Wendel-Vos, Droomers, Kremers, Brug, & van Lenthe, 2007), and the findings of this research show that people with dementia are little different. They enjoy physical activity more when they participate with others, and they recognize their need for companionship to help them maintain an active lifestyle (Chong et al., 2012).

The participants in this study also identified similar barriers. Like other older people, they described how health problems and environmental factors limited their ability to be as active as they would like (Schutzer & Graves, 2004).

The similarities with the research on older adults in general serve as a reminder that it is important to see people with dementia in the full context of their lives, and to not overstate the influence of the illness. Dementia may not constitute a radical disruption in how people frame their needs and desires around physical activity.

This is not to say, however, that the dementia did not matter. There were certainly indications in the interviews that dementia was influential in some of the participants’ decisions and actions around physical activity. While they did not talk about their impairment directly, they did allude to how problems with their thinking interfered with their physical activity and felt that they were less active because they lacked motivation. Research has shown that predictable and familiar activities present fewer barriers for people with dementia, which may allow them to retain a sense of purpose and control (Nygård, 2004; Roach & Drummond, 2014). Findings from the current study further suggest that with the kinds of memory and motivational challenges they experience, people with dementia appreciate the structure and focus that physical activity provides. The finding that participants valued being active in the outdoors is particularly noteworthy, aligning as it does with research suggesting that feeling connected to nature is important for people with dementia (Gibson, Chalfont, Clarke, Torrington, & Sixsmith, 2007; Olsson, Lampic, Skovdahl, & Engström, 2013). Outdoor physical activity may be one way they can achieve this.

Given the challenges they faced, the participants in this study made certain adjustments, and most found that walking was becoming their preferred activity. For some, this was an extension of a long-standing practice, while for others, it constituted a significant shift in their daily routine. Regardless, walking (sometimes alone, but more often with others) was becoming an increasingly important way for them to be active and engaged in the world.

This aligns with previous research showing how people with dementia respond through changes in general activity (Menne et al., 2002; Phinney et al., 2007; Phinney et al., 2013). It resonates also with findings from a recent meta-synthesis of older adults’ perceptions of mobility and its importance in their lives. Analyzing the results of 12 qualitative studies, Goins et al. (2014) found that mobility contributes to a sense of self and personal feelings of wholeness, and that being adaptable in the face of mobility challenges was of primary importance to older people. These findings provide context for understanding the potential significance of the current study showing that walking becomes increasingly meaningful for people with dementia, and it is something that they can do with others as the need emerges. As such, it is an activity that provides opportunities for engagement and social connection, both of which are important for supporting well-being in later life (Adams, Leibbrandt, & Moon, 2011).

### Practice Implications

As an interpretive description analysis, the purpose here was not only to describe subjective experiences of physical activity of people with dementia but also to situate the findings in terms of how they might inform practice. Findings from this small-scale study may not be broadly generalizable, but they do offer a better understanding that provides guidance for nursing practice in two key areas. First is the issue of “wandering.” Traditionally regarded as a behavior problem in long-term care settings, there have been calls in the nursing literature to re-conceptualize wandering as meaningful activity (Dewing, 2006), and this research provides some much-needed empirical evidence to support this effort. The subjective meaning of physical activity, and walking in particular, for people with mild to moderate dementia may provide important clues as to its significance for those with more advanced disease who “wander.” Rather than being considered as an irrational behavioral disturbance, it may be better understood as a way that people bring focus and structure when there is nothing else to do, or a way they can engage socially in the world around, or perhaps “wandering” reflects a simple need for enjoyable activity that makes people feel better. These are indeed important meanings, which may provide some direction for nurses who are seeking improved ways to support people with advanced dementia who are physically active and who want to walk.

Second is the issue of program development to support physical activity for people with dementia. While there remains a need for research to define appropriate guidelines for physical activity of older people with dementia (Hughes et al., 2011), this study suggests that walking programs for community dwelling people with dementia would be well received by many, especially if they were designed as group activities that were offered outdoors. A few studies have shown there to be some benefit of walking programs in residential care (e.g., Cott, Dawson, Sidani, & Wells, 2002; Taylor et al., 2003; Venturelli, Scarsini, & Schena, 2011), but the idea is only beginning to be translated for people with dementia living in the community (e.g., Mapes & Hine, 2011; Phinney, Baumbusch, O’Connor, Purves, & Kelson, 2014). A recent scoping study indicated that further research in this area is clearly warranted (Bowes, Dawson, Jepson, & McCabe, 2013). This study has taken a step in that direction by providing preliminary evidence to support future development and evaluation of community-based walking programs for people with dementia, thus ensuring that their subjective experiences and needs are taken into consideration.

## Conclusion

This study has explored the experiences of people with dementia who identify physical activity as an important part of their daily lives. The experiences of those who did not talk about physical activity in their interviews may be different—this is an inevitable limitation of the study design. Nevertheless, the findings offer important insight into the *potential* meanings of physical activity for people with dementia and provide some beginning directions for considering how to create better supports for these individuals and their families.

Taken as a whole, the findings show that like other older people, many people with dementia want to feel fit and healthy, and find physical activity an enjoyable way to spend time. This suggests that in planning supports for people with dementia, it is important to learn what they have always found to be meaningful and to take into consideration the place that physical activity has held for them in the past. Dementia does not necessarily change those values and desires.

That said, people with dementia may be well aware that it is not as easy as before and that while they are physically able, they feel their cognitive abilities make it difficult for them to be as active as they would like. Their preference for simple activities like walking and to be in the company of others may be interpreted as effective adaptations that allow them to remain involved in physical activity, which supports not only their sense of continuity and self but also their feelings of security and confidence.

In summary, while there is research showing the objective benefits of physical activity for people with dementia, this study is one of the very few that sheds light on the subjective benefits, while highlighting the particular significance of walking. In so doing, it contributes insights that have the potential to shape interventions aimed at helping people with dementia maintain physical activity as a part of their everyday lives.
